# Cardiac rehabilitation for patients with obesity: lessons learned from the OPTICARE XL trial

**DOI:** 10.1007/s12471-023-01832-w

**Published:** 2023-11-20

**Authors:** Iris den Uijl, Madoka Sunamura, Rutger M. W. Brouwers, Henk J. Stam, Eric Boersma, Rita J. G. van den Berg-Emons, Nienke ter Hoeve

**Affiliations:** 1 Department of Rehabilitation Medicine, Erasmus University Medical Centre, Rotterdam, The Netherlands; 2 Capri Cardiac Rehabilitation, Rotterdam, The Netherlands; 3 Department of Cardiology, Máxima Medical Centre, Eindhoven/Veldhoven, The Netherlands; 4 Department of Cardiology, Thoraxcenter, Erasmus University Medical Centre, Rotterdam, The Netherlands

**Keywords:** Cardiac rehabilitation, Obesity, Lifestyle, Cardiovascular disease, Atrial fibrillation

## Abstract

Obesity is a known and commonly encountered risk factor for the development of cardiac diseases. Patients with cardiac diseases who also have obesity do not benefit optimally from standard cardiac rehabilitation (CR) programs. Exercises performed during CR are not the best fit for patients with obesity and counselling sessions often do not address their specific needs. OPTICARE XL is the first large multicentre randomised controlled trial to investigate the added value of a dedicated one-year CR program specifically designed for patients with obesity and integrated in daily practice. The short-term effects on body weight and physical activity were promising and patients with obesity experienced the program as highly desirable. However, the OPTICARE XL CR program did not show long-term added value compared with standard CR on health-related quality of life, psychosocial well-being, body weight, physical activity and physical fitness, nor on costs. The current article offers an overview of the background of this trial and discusses the most important results of the OPTICARE XL trial and the reasons behind the unanticipated long-term outcomes. Furthermore, it offers recommendations for future research and how to redesign the OPTICARE XL CR program to expand the short-term results.

## What’s new?

- More than a third of cardiac patients suffer from obesity and standard cardiac rehabilitation (CR) programs are suboptimal in this increasing patient population. To address this issue, the OPTICARE XL CR program was developed to provide a dedicated cardiac rehabilitation approach for patients with obesity.
- Although we did not observe long-term effectiveness of the OPTICARE XL CR program, the short-term effects on body weight and physical activity were promising and patients with obesity experienced the program as highly desirable.
- The after-care phase of the OPTICARE XL CR program needs to be redesigned to expand the short-term effects.

## Introduction

Obesity is one of the risk factors for the development of cardiac diseases and has reached epidemic proportions [1]. In Europe, 38% of patients with cardiovascular diseases (CVD) are obese [2]. Furthermore, various studies have shown that obesity is associated with an increased prevalence of atrial fibrillation (AF) [3, 4]. In people with obesity, cardiovascular risk factors such as high cholesterol, hypertension, diabetes, unhealthy dietary habits and an inactive lifestyle are more profound [1]. Therefore, in patients with both cardiac disease and obesity, adoption of a healthy lifestyle is even more crucial to prevent new cardiovascular events, which are associated with high rates of healthcare utilisation and costs [5]. Exercise-based cardiac rehabilitation (CR) is an effective secondary prevention strategy for a broad range of patients with cardiac diseases [5, 6]. CR programs focus on physical and psychosocial recovery by targeting cardiovascular risk factors, restoring psychosocial balance and facilitating the adoption of a healthy lifestyle. Nevertheless, it has been suggested that standard CR programs may not be optimal for patients with obesity.

This overview provides a comprehensive summary of the current knowledge on the effectiveness of standard CR programs for patients with obesity. Furthermore, it summarises and highlights key findings and lessons learned from the outcomes of the OPTICARE XL multicentre randomised controlled trial, which were published previously in a series of papers [7–9]. The OPTICARE XL study evaluated the effectiveness of a unique dedicated CR program designed specifically for patients with both CVD or AF and obesity. Finally, this overview offers recommendations for future research to better understand and address the needs of this growing target population.

## Patients with obesity in standard CR programs

CR programs typically involve an exercise program, psychological counselling and educational sessions [5, 6, 10]. CR programs generally last for 6–12 weeks and the main goals are to increase the patient’s physical fitness, reach a stable psychosocial status, manage cardiovascular risk factors and achieve a heart-healthy lifestyle [5, 6, 10]. CR has shown to be effective in improving health-related quality of life (HRQOL) and physical fitness, and in reducing the risk of mortality and hospital readmissions for a broad range of cardiac patients in a cost-effective way [11–15].

However, evidence suggests that standard CR programs may not be optimal for patients with obesity. This group typically presents with a more unfavourable cardiovascular risk profile than cardiac patients with a normal weight, including a higher prevalence of hypertension and dyslipidaemia, poorer physical fitness, a less favourable physical activity and a sedentary behaviour profile [16–18]. Therefore, especially in cardiac patients with obesity, achieving goals such as losing weight, increasing physical activity and improving physical fitness is crucial. [5, 19, 20] Nevertheless, these lifestyle goals are often not met during participation in standard CR programs. Despite studies indicating that a weight loss of 5–10% is associated with reduced cardiovascular risk [21–23], it is estimated that over 80% of patients with obesity fail to reach this target [24]. Furthermore, changes in physical fitness and physical activity levels are only small and clinically relevant thresholds are often not achieved [18, 25]. For example, obese patients participating in CR programs generally only manage around 5500 steps per day [18], while a minimum of 6500 steps/day is recommended to prevent disease progression [26].

The findings highlighted above suggest that CR programs should be adapted to meet the needs of patients with obesity. Previous research also emphasises the need for a CR program designed specifically for this patient group [24, 25]. The predominantly weight-bearing exercises often applied in CR programs might not be the best fit for the obese patient population, because of the higher risk for musculoskeletal symptoms [27, 28]. Previous research has pointed at benefits of a combination of endurance training with mainly non-weight-bearing exercises (e.g. on a cycle or rowing ergometer) and resistance training, aimed at expansion to activities with a higher caloric energy expenditure [27, 28]. In addition to exercise training, the general counselling on a healthy lifestyle provided during standard CR might also not fit the needs of obese patients [25, 29]. Patients with obesity generally receive counselling with patients of other body mass index (BMI) classes while the needs of obese patients in terms of healthy food choices, losing body weight and adopting an active lifestyle are likely to be different. Peer support can play a significant role in behaviour change and could be enhanced by offering a CR program in small groups exclusively for patients with obesity. This would provide a supportive environment where patients can share their experiences and challenges, creating a sense of community and promoting long-term adherence to healthy behaviours. Additionally, a longer time period than the usual 3 months of standard CR is preferred for achieving lasting behavioural changes [30]. In coaching sessions, the use of behaviour change techniques such as self-monitoring, goal setting and planning are recommended to help patients adapt to a healthier lifestyle in their own environment [31–33]. Several tools and methods could be applied to facilitate this behaviour change. Previous research has, for instance, shown the benefits of using activity trackers to stimulate physical activity and prevent sedentary behaviour [32–34].

## The OPTICARE XL trial: outcomes and lessons learned

### The OPTICARE XL CR program

In the OPTICARE XL randomised controlled trial, the long-term effectiveness of a new, dedicated CR program designed for patients with obesity (OPTICARE XL CR) was investigated in three large CR centres in The Hague, Eindhoven and Rotterdam, the Netherlands. A total of 201 patients with documented coronary artery disease (CAD) or nonvalvular atrial fibrillation (AF) and who had a BMI ≥ 30 kg/m^2^ were included and randomised to OPTICARE XL CR (*n* = 102) or standard CR (*n* = 99). Standard CR was offered based on Dutch and European guidelines [5, 6], and included a 6–12-week exercise program in combination with information sessions on healthy lifestyle, psychosocial recovery and cardiovascular risk factors. OPTICARE XL CR took 1 year to complete and was performed in small peer groups consisting of 6–8 patients with obesity. During the first 3 months, patients received exercise sessions, including a combination of non-weight-bearing aerobic exercises and resistance training. In addition to usual information sessions and optional modules as offered in the standard CR program, patients received two group coaching modules: the *Healthy Weight *and the *Active Lifestyle *module. These modules were led by dieticians, physical therapists and behavioural change specialists and aimed to guide patients with obesity towards a healthier lifestyle by combining several behavioural change techniques, such as self-monitoring, goal setting, planning, receiving feedback, identifying barriers and developing plans for relapse prevention. [31–33]. Patients then joined a 9-month after-care program, which consisted of six behavioural booster sessions and access to a group chat. During the entire program, patients were offered the use of an activity tracker (Garmin VivoSmart HR XL). Differences between the characteristics of OPTICARE XL CR and standard CR are outlined in Tab. 1.

**Table 1:** Characteristics of OPTICARE XL CR and standard CR

	OPTICARE XL CR	Standard CR
Duration of program	Part I: 12 weeks	6–12 weeks
	Part II: 9‑month after-care program	No after-care program
Participating patients	Only patients with obesity	Patients with and without obesity
	Group size: 6–8 patients	Group size 10–25 patients
Exercise program	60–90 min supervised exercise sessions (twice a week during part 1)	60–90 min supervised exercise sessions (twice a week)
	Combination of endurance and resistance training	Mainly endurance training
	Designed for patients with obesity	Designed for patients of all BMI classes
Counselling sessions	General group information sessions on lifestyle and risk factors	General group information sessions on lifestyle and risk factors
	Facultative counselling in smoking cessation, stress management or healthy diet coaching on indication	Facultative counselling in smoking cessation, stress management or healthy diet coaching on indication
	Additional intensive coaching during part I by means of two modules (in total 16 sessions), focussing on (1) healthy diet and (2) active lifestyle. Behavioural change techniques were applied, such as self-monitoring, goal setting	No additional coaching
	Six booster sessions during part II	
Technology	An activity tracker was provided to promote an active lifestyle during part I and part II	No use of additional technologies
	Voluntary participation in a group chat in part II	

*CR* cardiac rehabilitation, *BMI* body mass index

### Outcomes of the OPTICARE XL trial

**Fig. 1:**
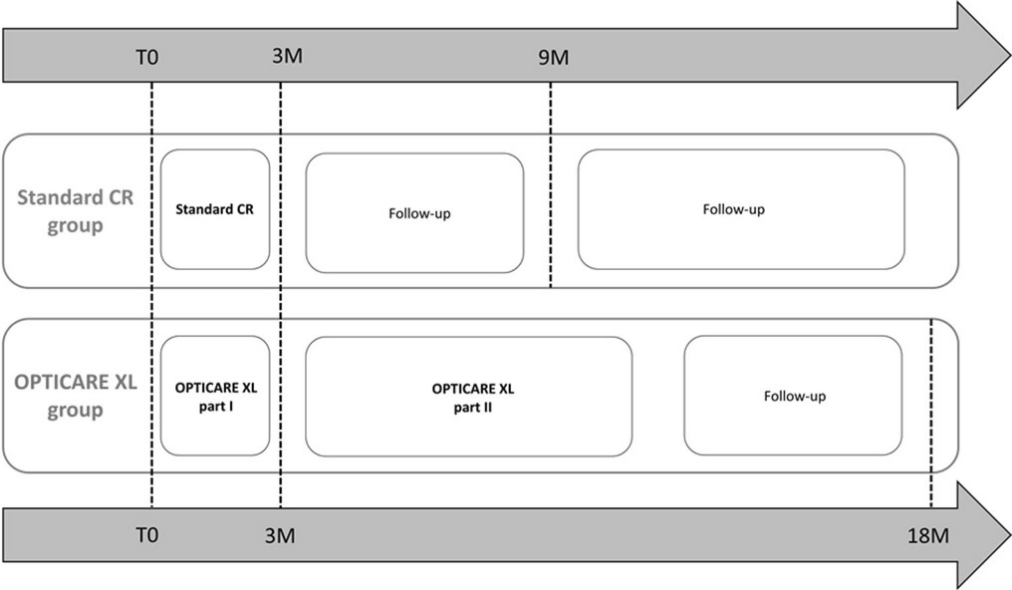
Study design

The effects of OPTICARE XL CR versus standard CR were studied according to a broad range of outcomes, including weight, physical fitness, physical activity and HRQOL, which were measured up to 6 months after completion of either program (Fig. 1) and are summarised in Tab. 2. These results were described in a series of three papers: one on HRQOL and psychosocial well-being [7], one on physical outcomes [8] and one on cost-effectiveness of OPTICARE XL CR [9]. Overall, the results of the OPTICARE XL trial did not support our hypothesis that the OPTICARE XL CR program would be more effective than standard CR in patients with obesity in the long term. Although a statistically significant larger weight loss (−3.6 kg versus −1.8 kg, *p* = 0.002) and a numerically larger improvement in steps/day (+880 versus +481, *p* = 0.324) was observed in patients allocated to OPTICARE XL CR during the first 3 months, these differences between study groups disappeared in the period thereafter [8]. For body weight, a loss of 5–15% in a 6-month period is realistic and recommended [35, 36]. Patients in the OPTICARE XL CR group lost on average 3.5% body weight in the first 3 months, which seems sufficient given the time period. However, patients allocated to both OPTICARE XL CR and standard CR showed a mean weight loss of only 2.5 kg (3.5%) 6 months after completion of either program with no differences between groups. With regard to physical activity, the higher average number of steps in the patients randomised to OPTICARE XL CR seen in the first 3 months still did not reach or exceed the clinically relevant threshold of 6500 steps/day [26]. Nevertheless, we observed a significant mean improvement of 880 steps/day in the OPTICARE XL CR group. Although this improvement was not significantly larger than the improvement of 481 steps/day in the standard CR group, the numerical difference does indicate a potential added value of OPTICARE XL CR on physical activity. Unfortunately, these improvements partly disappeared over the longer term.

**Table 2:** Summary of OPTICARE XL outcomes described in this paper

Outcome	Long-term difference* between OPTICARE XL and standard CR	Short-term difference* between OPTICARE XL and standard CR
Weight loss (kg)	No significant difference between groups	Significant difference: −3.6 in OPTICARE XL CR vs −1.8 in standard CR, *p* = 0.002
Physical activity (steps per day)	No significant difference between groups	Numerically larger improvement in OPTICARE XL CR: +880 vs +481, *p* = 0.324
Physical fitness (6-minute walk test)	No significant difference between groups	No significant difference between groups
Health-related quality of life (MacNew questionnaire)	No significant difference between groups	No significant difference between groups
Cost-effectiveness	NA	No significant difference between groups

*Long-term difference = 6 months after completion of CR. Short-term difference = 3 months after the start of CR

*CR* cardiac rehabilitation, *N/A* not available

In terms of physical fitness, measured as total distance on the 6‑minute walk test, we observed significant improvements within both study groups; however, no significant difference between patients allocated to OPTICARE XL CR or standard CR was observed [8]. The improvements on the 6‑minute walk test exceeded the clinically relevant improvement of 25 meter within both study groups [37]. For HRQOL, measured with the MacNew Heart Disease Health-Related Quality of Life Instrument, we observed relatively high baseline levels [7]. Nevertheless, we still found additional, but equal, improvements in both groups. This indicates that the majority of cardiac patients with obesity do not require further attention to enhance HRQOL.

An economic evaluation of OPTICARE XL CR was performed to assess cost-effectiveness. Economic evaluations are designed to inform policy makers and healthcare physicians on the costs and health benefits of a new intervention. This study showed no differences in health gains between patients allocated to OPTICARE XL or standard CR, and no overall additional cost savings [9]. A breakdown of costs showed higher intervention-related costs in patients randomised to OPTICARE XL, but medical-related costs and indirect costs were lower than in patients allocated to standard CR. While from an economic point of view it is most logical to discourage rehabilitation centres from offering such an extensive and therefore more expensive CR program, it could also be argued that a program which suits patients with obesity better might cause higher referral rates of obese patients to CR as well as lower drop-out numbers during the program.

### Lessons learned

Although the short-term effects on body weight and physical activity were promising, we did not observe long-term effectiveness of the OPTICARE XL CR program. The unexpected long-term results can be attributed to various factors. Notably, a considerable proportion (393 out of 698 patients) declined participation in the OPTICARE XL trial, primarily citing motivational factors. Consequently, our participant pool might have predominantly consisted of highly motivated individuals. This is evidenced by the relatively favourable outcomes in our control group regarding HRQOL and physical fitness, making it harder to demonstrate the additional benefit of the OPTICARE XL program. Nevertheless, since the outcomes with regard to weight loss and physical activity are still disappointing, this patient selection cannot fully explain the unanticipated outcomes. Another contributing factor could be the drop-out of patients, with approximately one third of patients randomised to OPTICARE XL visiting less than 75% of all sessions. Nonetheless, even among patients who adhered to the program, the outcomes did not demonstrate substantial long-term improvement. This suggests that the disappointing outcomes are mainly due to the current program being suboptimal. Literature suggests that short-term outcomes with regard to weight loss could probably be enlarged by stimulating a lower energy diet and offering higher caloric exercises, such as aerobic interval training at peak heart rate [24]. Nevertheless, these kinds of diets and exercises are much harder to maintain in the longer term. Furthermore, since short-term results with regard to weight loss and physical activity were promising, redesign mainly seems needed in the after-care phase. During interview sessions with patients, we gathered advice on how to redesign the OPTICARE XL CR program to expand the initial added value. First, it was evident that the redesign should at least include a reconsideration of the frequency of sessions in the transition from the first part to the after-care phase of the program. This is confirmed by the literature, suggesting that a higher number of sessions in the first 6 months of an intervention can lead to further weight loss [38]. Increasing the frequency of face-to-face sessions is expected to be costly and a burden for patients and healthcare providers, and therefore other ways of providing after-care should be considered such as telerehabilitation. Cardiac telerehabilitation was shown to be at least as effective as standard CR in non-obese patients [39]. Secondly, when redesigning the OPTICARE XL CR program, all stakeholders need to be included. Besides CR healthcare professionals, researchers and patients, also healthcare professionals from primary care, partners of patients, and lifestyle coaches should be involved. Thirdly, active referral to contacts within the network of CR centres who focus on a combination of diet and physical activity might be advised, such as the Dutch combined lifestyle interventions (CLI) which are offered within the patient’s home environment. Although overall these CLI programs have proved to result in only a mild weight reduction, it was shown that programs with a stronger focus on physical activity and a higher number of meetings are capable of resulting in a long-term weight loss above 5% [38].

## Recommendations for future research

There is a significant demand for specialised care for cardiac patients with obesity. To our knowledge, the OPTICARE XL trial is the first study to investigate the effectiveness and cost-effectiveness of a CR program specifically designed for patients with obesity with a long follow-up period, in a multicentre randomised controlled trial. While the OPTICARE XL CR program did not demonstrate added value in the long-term as compared with standard CR, the short-term results on body weight and physical activity were encouraging. Identifying the most effective CR program for this growing patient population continues to be a challenge. Further research is needed to determine the optimal combination of exercise, counselling and behaviour change techniques that will promote sustained weight loss and long-term cardiovascular health in this population.

To enhance the effectiveness of CR programs for patients with obesity, future studies could help in further personalisation of the program by investigating factors associated with goal achievement, including identifying which patients are able to reach their goals and which patients are not. In addition, further utilising qualitative research methods such as interviews or focus groups can provide valuable insights into the preferences and needs of patients with regard to CR programs. After redesigning the investigated program, a new study is recommended to find out whether the revised version has better results in markers of success of CR. Although a randomised controlled trial design is known as the most solid study design, other study designs should also be considered, for example a cohort study, possibly by using a national registry. By applying such a study design, also less motivated patients are likely to be included and iterations to the CR program can more easily be implemented and tested.

## Conclusion

There is a high need for specialised care for cardiac patients with obesity. Although we did not observe long-term effectiveness of a program developed for these patients, the short-term effects on body weight and physical activity were promising and patients with obesity experienced the program as highly desirable. Future CR programs should be adapted to find the best fit for this growing target population.

## References

[1] Obesity Collaborators GBD (2017). Health effects of overweight and obesity in 195 countries over 25 years. New Engl J Med.

[2] Kotseva K, De Backer G, De Bacquer D (2019). Lifestyle and impact on cardiovascular risk factor control in coronary patients across 27 countries: results from the European society of cardiology ESC-EORP EUROASPIRE V registry. Eur J Prev Cardiol.

[3] Wanahita N, Messerli FH, Bangalore S (2008). Atrial fibrillation and obesity—results of a meta-analysis. Am Heart J.

[4] Zhuang J, Lu Y, Tang K, Peng W, Xu Y (2013). Influence of body mass index on recurrence and quality of life in atrial fibrillation patients after catheter ablation: a meta-analysis and systematic review. Clin Cardiol.

[5] Piepoli MF, Hoes AW, Agewall S (2016). European Guidelines on cardiovascular disease prevention in clinical practice: The Sixth Joint Task Force of the European Society of Cardiology and Other Societies on Cardiovascular Disease Prevention in Clinical Practice (constituted by representatives of 10 societies and by invited experts). Developed with the special contribution of the European Association for Cardiovascular Prevention &amp; Rehabilitation (EACPR). Eur Heart J.

[6] Revalidatiecommissie NVVC/ NHS en projectgroep PAAHR. Multidisciplinary guidelines cardiac rehabilitation on 2011. Multidisciplinaire Richtlijn Hartrevalidatie, Vol. 2011. Utrecht: Nederlandse Vereniging voor Cardiologie; 2011.

[7] Den Uijl I, Ter Hoeve N, Sunamura M (2023). Cardiac rehabilitation designed for patients with obesity: OPTICARE XL RCT results on health-related quality of life and psychosocial well-being. Disabil Rehabil.

[8] Den Uijl I, Van den Berg-Emons RJ, Sunamura M (2023). Effects of a dedicated cardiac rehabilitation program for patients with obesity on body weight, physical activity, sedentary behaviour and physical fitness: the OPTICARE XL randomized controlled trial. Phys Ther J.

[9] Visser L, den Uijl I, Redekop W (2023). Cost-effectiveness of a cardiac rehabilitation program specifically designed for patients with obesity within the OPTICARE XL randomized controlled trial. Arch Phys Med Rehabil.

[10] Balady GJ, Williams MA, Ades PA, et al. Core components of cardiac rehabilitation/secondary prevention programs: 2007 update: A scientific statement from the American Heart Association Exercise, Cardiac rehabilitation, and Prevention Committee, the Council on Clinical Cardiology; the Councils on Cardiovascular Nursing, Epidemiology and Prevention, and Nutrition, Physical Activity, and Metabolism; and the American Association of Cardiovascular and Pulmonary Rehabilitation. Circ. 2007;115:2675–82. 10.1161/CIRCULATIONAHA.106.180945 17513578

[11] Anderson L, Thompson DR, Oldridge N (2016). Exercise-based cardiac rehabilitation for coronary heart disease. Cochrane Database Syst Rev.

[12] De Vries H, Kemps HM, van Engen-Verheul MM, Kraaijenhagen RA, Peek N (2015). Cardiac rehabilitation and survival in a large representative community cohort of Dutch patients. Eur Heart J.

[13] Francis T, Kabboul N, Rac V (2019). The effect of cardiac rehabilitation on health-related quality of life in patients with coronary artery disease: a meta-analysis. Can J Cardiol.

[14] Lavie CJ, Milani RV (2011). Cardiac rehabilitation and exercise training in secondary coronary heart disease prevention. Prog Cardiovasc Dis.

[15] Takura T, Ebata-Kogure N, Goto Y, et al. Cost-effectiveness of cardiac rehabilitation in patients with coronary artery disease: A meta-analysis. Cardiol Res Pract. 2019;4:2019:1840894. 10.1155/2019/1840894 PMC6589196 31275640

[16] NCD Risk Factor Collaboration. Trends in adult body-mass index in 200 countries from 1975to 2014: a pooled analysis of 1698 population-based measurement studies with 19.2 million participants. Lancet. 2016;387:1377–96. 10.1016/S0140-6736(16)30054-X PMC7615134 27115820

[17] Gunstad J, Luyster F, Hughes J (2007). The effects of obesity on functional work capacity and quality of life in phase II cardiac rehabilitation. Prev Cardiol.

[18] Den Uijl I, Ter Hoeve N, Sunamura M (2021). Physical Activity and Sedentary Behavior in Cardiac Rehabilitation: Does Body.

[19] Lavie CJ, Pandey A, Lau DH, Alpert MA, Sanders P (2017). Obesity and atrial fibrillation prevalence, pathogenesis, and prognosis: effects of weight loss and exercise. J Am Coll Cardiol.

[20] Ekelund U, Tarp J, Steene-Johannessen J (2019). Dose-response associations between accelerometry measured physical activity and sedentary time and all cause mortality: systematic review and harmonised meta-analysis. Br Med J.

[21] Ades PA, Savage PD, Harvey-Berino J (2010). The treatment of obesity in cardiac rehabilitation. J Cardiopulm Rehabil Prev.

[22] Manzoni GM, Villa V, Compare A (2011). Short-term effects of a multi-disciplinary cardiac rehabilitation program on psychological well-being, exercise capacity and weight in a sample of obese in-patients with coronary heart disease: a practice-level study. Psychol Health Med.

[23] Wing RR, Lang W, Wadden TA (2011). Benefits of modest weight loss in improving cardiovascular risk factors in overweight and obese individuals with type 2 diabetes. Diabetes Care.

[24] De Bacquer D, Jennings CS, Mirrakhimov E (2022). Potential for optimizing management of obesity in the secondary prevention of coronary heart disease. Eur Heart J Qual Care Clin Outcomes.

[25] Ades PA, Savage PD (2021). The Treatment of Obesity in Cardiac Rehabilitation: a review and practical recommendations. J Cardiopulm Rehabil Prev.

[26] Ayabe M, Brubaker PH, Dobrosielski D (2008). Target step count for the secondary prevention of cardiovascular disease. Circ J.

[27] Ho SS, Dhaliwal SS, Hills AP, Pal S (2012). The effect of 12 weeks of aerobic, resistance or combination exercise training on cardiovascular risk factors in the overweight and obese in a randomized trial. Bmc Public Health.

[28] Schjerve IE, Tyldum GA, Tjønna AE (2008). Both aerobic endurance and strength training programs improve cardiovascular health in obese adults. Clin Sci.

[29] De Dirk B, Dallongeville J, Heidrich J (2010). Management of overweight and obese patients with coronary heart disease across Europe. Eur J Prev Cardiol.

[30] Ter Hoeve N, Sunamura M, Stam HJ (2018). Effects of two behavioral cardiac rehabilitation interventions on physical activity: a randomized controlled trial. Int J Cardiol.

[31] Aldcroft SA, Taylor NF, Blackstock FC, O’Halloran PD (2011). Psychoeducational rehabilitation for health behavior change in coronary artery disease: a systematic review of controlled trials. J Cardiopulm Rehabil Prev.

[32] Chase J-AD (2011). Systematic review of physical activity intervention studies after cardiac rehabilitation. J Cardiovasc Nurs.

[33] Ferrier S, Blanchard CM, Vallis M, Giacomantonio N (2011). Behavioural interventions to increase the physical activity of cardiac patients: a review. Eur J Prev Cardiol.

[34] Braakhuis HE, Berger MA, Bussmann JB (2019). Effectiveness of healthcare interventions using objective feedback on physical activity: A systematic review and meta-analysis. J Rehabil Med.

[35] Jensen MD, Ryan DH, Apovian CM, et al. 2013 AHA/ACC/TOS guideline for the management of overweight and obesity in adults: a report of the American College of Cardiology/American Heart Association Task Force on Practice Guidelines and The Obesity Society. J Am Coll Cardiol. 2014;63(25 Part B):2985–3023. 10.1016/j.jacc.2013.11.004 24239920

[36] Yumuk V, Tsigos C, Fried M (2015). European guidelines for obesity management in adults. Obes Facts.

[37] Gremeaux V, Troisgros O, Benaïm S (2011). Determining the minimal clinically important difference for the six-minute walk test and the 200-meter fast-walk test during cardiac rehabilitation program in coronary artery disease patients after acute coronary syndrome. Arch Phys Med Rehabil.

[38] Stiggelbout M, De Gecombineerde Leefstijlinterventie RH (2023). Goed Op Weg!? Ned Tijdschr Leefstijlgeneesk.

[39] Brouwers RWM, van Exel HJ, van Hal JMC (2020). Cardiac telerehabilitation as an alternative to centre-based cardiac rehabilitation. Neth Heart J.

